# Supplementary material for: Stray Dogs and Public Health: Population Estimation in Punjab, India
Source: Vet Sci. 2022 Feb 10;9(2):75. doi: 10.3390/vetsci9020075 (PMC8878280; doi:10.3390/vetsci9020075)
Supplement: Supplementary file 1 [file vetsci-09-00075-s001.zip › vetsci-1568031-supplementary.pdf]

Table S1: The adult human population (Census India 2011) and the residential area of selected villages and wards included in a survey to determine the dog population of the Punjab state of India.

Name of Village/Ward	District	Adult Human Population	Residential Area (in km²)
		**Rural**	
Galib Ran Singh	Ludhiana	929	0.07
Pipli	Faridkot	2808	0.37
Jodhpur	Barnala	2728	0.27
Rauli	Hoshiarpur	753	0.08
Mangewala	Moga	1836	0.15
Karahal Kalan	Kapurthala	583	0.05
Deugarh	Patiala	836	0.08
Mander	Mansa	1986	0.16
Rali Ali Kalan	Gurdaspur	950	0.09
Mahdian	Fateghrsahib	1211	0.1
Rangian	Ropar	1150	0.09
Gobindpura	Bathinda	4822	0.42
Kot Kalan	Jalandher	663	0.1
Khoba	Pathankot	792	0.03
Gobindgarh	Fazilka	3153	0.4
Jethuwal	Amritsar	2985	0.23
Bugar	Sangrur	1930	0.07
Haraj	Muktsar	1159	0.16
Nabipur	Tarntarn	999	0.12
Kheda	Mohali	772	0.07
Tut	Firozpur	1656	0.16
Sodhian	Nawanshehar	755	0.06

**Urban**			
Bhagta Bhai ka ward :8	Bathinda	1136	0.05
Bhagta Bhai ka ward :1	Bathinda	964	0.14
Balachaur ward :4	Nawanshehar	1066	0.2
Balachaur ward :5	Nawanshehar	1136	0.05
Bathinda ward :9	Bathinda	3695	0.37
Bathinda ward :11	Bathinda	4370	0.49
Zeera ward: 15	Firozpur	1461	0.17
Zeera ward: 16	Firozpur	1732	1.29
Ludhiana ward: 62	Ludhiana	6415	0.47
Ludhiana ward: 63	Ludhiana	16067	1.21
**Overall**			
Punjab	Rural	12201170	1857.24
	Urban	7315518	1139.39
	Industrial		60.01

Table S2: Total number of stray dogs estimated in different village(s)/ward(s) in Punjab state of India (using *Lincoln-Petersen's formula with Chapman's correction*)

Name of the village/ward	District	Type of dog	n1	n2	m	Stray dog count per village/ward (95% CI)	Stray Dog Population /km2 (95% CI)	Stray Dogs/1000 adult humans
Galib Ran Singh	Ludhiana	Adult Male	13	11	10	14 (13, 15)	204 (186, 214)	15 (14, 16)
		Adult female	9	10	6	15 (11, 18)	210 (157, 257)	16 (12, 19)
		Total adult	22	21	16	29 (24, 32)	411 (343, 457)	31 (26, 34)
		Young male	3	4	3	4 (4, 4)	57 (57, 57)	4 (4, 4)
		Young female	2	1	1	2 (2, 2)	29 (29, 29)	2 (2, 2)
		Total young	5	5	4	6 (5, 7)	89 (71, 100)	7 (5, 8)
		Total male	16	15	13	18 (17, 20)	263 (243, 286)	20 (18, 22)
		Total female	11	11	7	17 (13, 21)	243 (186, 300)	18 (14, 23)
		**Total dogs**	**27**	**26**	**20**	35 (31, 39)	500 (443, 557)	38 (33, 42)
Pipli	Faridkot	Adult Male	18	21	12	31 (25, 37)	84 (68, 100)	11.09 (9, 13)
		Adult female	17	18	13	23 (20, 26)	63 (54, 70)	8.34 (7, 9)

		Total adult	35	39	25	54 (48, 61)	147 (130, 165)	19.37 (17, 22)
		Young male	5	4	2	9 (5, 13)	24 (14, 35)	3.21 (2, 5)
		Young female	3	5	2	7 (4, 10)	19 (11, 27)	2.49 (1, 4)
		Total young	8	9	4	17 (8, 26)	46 (22, 70)	6.05 (3, 9)
		Total male	23	25	14	41 (32, 49)	110 (86, 132)	14.46 (11, 17)
		Total female	20	23	15	31 (26, 35)	82 (70, 95)	10.86 (9, 12)
		**Total dogs**	**43**	**48**	**29**	71 (62, 80)	192 (168, 216)	25.24 (22, 28)
Jodhpur	Barnala	Adult Male	21	19	14	28 (24, 32)	105 (89, 119)	10.38 (9, 12)
		Adult female	17	20	15	23 (21, 24)	84 (78, 89)	8.3 (8, 9)
		Total adult	38	39	29	51 (47, 55)	189 (174, 204)	18.7 (17, 20)
		Young male	3	4	2	6 (4, 8)	21 (15, 30)	2.08 (1, 3)
		Young female	6	5	4	7 (6, 9)	27 (22, 33)	2.71 (2, 3)
		Total young	9	9	6	13 (10, 17)	49 (37, 63)	4.87 (4, 6)
		Total male	24	23	16	34 (29, 39)	127 (107, 144)	12.57 (11, 14)
		Total female	23	25	19	30 (28, 33)	112 (104, 122)	11.07 (10, 12)
		**Total dogs**	**47**	**48**	**35**	64 (59, 70)	238 (219, 259)	23.58 (22, 26)

Rauli	Hoshiarpur	Adult Male	5	4	3	7 (5, 8)	81 (63, 100)	8.63 (7, 11)
		Adult female	6	5	4	7 (6, 9)	93 (75, 113)	9.83 (8, 12)
		Total adult	11	9	7	14 (11, 17)	175 (138, 213)	18.59 (15, 23)
		Young male	1	2	1	2 (2, 2)	25 (25, 25)	2.66 (3, 3)
		Young female	2	2	1	4 (2, 5)	44 (25, 63)	4.65 (3, 7)
		Total young	3	4	2	6 (3, 9)	71 (38, 113)	7.53 (4, 12)
		Total male	6	6	4	9 (7, 11)	110 (88, 138)	11.69 (9, 15)
		Total female	8	7	5	11 (8, 14)	138 (100, 175)	14.61 (11, 19)
		**Total dogs**	**14**	**13**	**9**	20 (16, 24)	250 (200, 300)	26.56 (21, 32)
Mangewala	Moga	Adult Male	16	18	13	22 (20, 25)	147 (250, 313)	12.02 (11, 14)
		Adult female	15	12	10	18 (16, 20)	119 (200, 250)	9.75 (9, 11)
		Total adult	31	30	23	40 (36, 44)	269 (450, 550)	21.97 (20, 24)
		Young male	4	3	3	4 (4, 4)	27 (50, 50)	2.18 (2, 2)
		Young female	3	4	2	6 (4, 8)	38 (50, 100)	3.09 (2, 4)
		Total young	7	7	5	10 (7, 12)	64 (88, 150)	5.27 (4, 7)
		Total male	20	21	16	25 (24, 29)	168 (300, 363)	13.71 (13, 16)
		Total female	18	16	12	24 (20, 27)	159 (250,	12.99 (11,

							338)	15)
		**Total dogs**	**38**	**37**	**28**	50 (46, 55)	334 (575, 688)	27.29 (25, 30)
Karahal Kalan	Kapurthala	Adult Male	8	6	4	12 (8, 15)	232 (160, 300)	19.9 (14, 26)
		Adult female	4	5	4	5 (5, 5)	100 (100, 100)	8.58 (9, 9)
		Total adult	12	11	8	16 (13, 19)	327 (260, 380)	28.01 (22, 33)
		Young male	2	3	1	5 (2, 8)	100 (40, 160)	8.58 (3, 14)
		Young female	3	4	3	4 (4, 4)	80 (80, 80)	6.86 (7, 7)
		Total young	5	7	4	9 (6, 11)	172 (120, 220)	14.75 (10, 19)
		Total male	10	9	5	17 (11, 23)	347 (220, 460)	29.73 (19, 39)
		Total female	7	9	7	9 (9, 9)	180 (180, 180)	15.44 (15, 15)
		**Total dogs**	**17**	**18**	**12**	25 (21, 29)	506 (420, 580)	43.41 (36, 50)
Devgarh	Patiala	Adult Male	13	10	7	18 (14, 23)	228 (175, 288)	21.83 (17, 28)
		Adult female	7	6	5	8 (7, 10)	104 (88, 125)	9.96 (8, 12)
		Total adult	20	16	12	26 (22, 31)	331 (275, 388)	31.65 (26, 37)

		Young male	2	2	2	2 (2, 2)	25 (25, 25)	2.39 (2, 2)
		Young female	4	3	3	4 (4, 4)	50 (50, 50)	4.78 (5, 5)
		Total young	6	5	5	6 (6, 6)	75 (75, 75)	7.18 (7, 7)
		Total male	15	12	9	20 (16, 23)	248 (200, 288)	23.68 (19, 28)
		Total female	11	9	8	12 (11, 14)	154 (138, 175)	14.75 (13, 17)
		**Total dogs**	**26**	**21**	**17**	32 (28, 36)	400 (350, 450)	38.28 (33, 43)
Mander	Mansa	Adult Male	21	18	17	22 (21, 23)	139 (131, 144)	11.19 (11, 12)
		Adult female	16	14	12	19 (17, 20)	116 (106, 125)	9.38 (9, 10)
		Total adult	37	32	29	40 (39, 43)	255 (244, 269)	20.54 (20, 22)
		Young male	3	4	3	4 (4, 4)	25 (25, 25)	2.01 (2, 2)
		Young female	3	3	2	4 (3, 6)	27 (19, 38)	2.18 (2, 3)
		Total young	6	7	5	8 (7, 10)	52 (44, 63)	4.19 (4, 5)
		Total male	24	22	20	26 (25, 28)	165 (156, 175)	13.28 (13, 14)
		Total female	19	17	14	23 (21, 25)	144 (131, 156)	11.58 (11, 13)
		**Total dogs**	**43**	**39**	**34**	49 (47, 52)	308 (294, 325)	24.82 (24, 26)

Rali Ali Kalan	Gurdaspur	Adult Male	6	7	3	13 (7, 19)	144 (78, 211)	13.68 (7, 20)
		Adult female	4	5	2	9 (5, 13)	100 (56, 144)	9.47 (5, 14)
		Total adult	10	12	5	23 (14, 32)	254 (156, 356)	24.03 (15, 34)
		Young male	6	3	2	8 (5, 12)	93 (56, 133)	8.77 (5, 13)
		Young female	2	2	2	2 (2, 2)	22 (22, 22)	2.11 (2, 2)
		Total young	8	5	4	10 (7, 13)	109 (78, 144)	10.32 (7, 14)
		Total male	12	10	5	23 (14, 32)	254 (156, 356)	24.03 (15, 34)
		Total female	6	7	4	10 (7, 13)	113 (78, 144)	10.74 (7, 14)
		**Total dogs**	**18**	**17**	**9**	33 (24, 42)	369 (267, 467)	34.95 (25, 44)
Mahdian	Fatehgarh Sahib	Adult Male	9	7	6	10 (9, 12)	104 (90, 120)	8.61 (7, 10)
		Adult female	4	6	3	8 (5, 10)	78 (50, 100)	6.4 (4, 8)
		Total adult	13	13	9	19 (15, 22)	186 (150, 220)	15.36 (12, 18)
		Young male	1	2	0	5 (0, 10)	50 (0, 100)	4.13 (0, 8)
		Young female	3	4	3	4 (4, 4)	40 (40, 40)	3.3 (3, 3)
		Total young	4	6	3	8 (5, 11)	78 (50, 110)	6.4 (4, 9)
		Total male	10	9	6	15 (11, 18)	147 (110, 180)	12.15 (9, 15)
		Total female	7	10	6	12 (10, 13)	116 (100,	9.55 (8, 11)

							130)	
		**Total dogs**	**17**	**19**	**12**	27 (22, 31)	267 (220, 310)	22.04 (18, 26)
Rangian	Rupnagar (Ropar)	Adult Male	11	12	8	16 (13, 19)	181 (144, 211)	14.2 (11, 17)
		Adult female	6	7	5	8 (7, 10)	93 (78, 111)	7.24 (6, 9)
		Total adult	17	19	13	25 (21, 29)	275 (233, 322)	21.49 (18, 25)
		Young male	1	1	1	1 (1, 1)	11 (11, 11)	0.87 (1, 1)
		Young female	3	3	2	4 (3, 6)	48 (33, 67)	3.77 (3, 5)
		Total young	4	4	3	5 (3, 7)	58 (33, 78)	4.57 (3, 6)
		Total male	12	13	9	17 (14, 19)	191 (156, 211)	14.96 (12, 17)
		Total female	9	10	7	13 (10, 15)	142 (111, 167)	11.09 (9, 13)
		**Total dogs**	**21**	**23**	**16**	30 (26, 34)	334 (289, 378)	26.14 (23, 30)
Gobindpura	Bathinda	Adult Male	23	21	17	28 (25, 31)	67 (60, 74)	5.88 (5, 6)
		Adult female	18	15	11	24 (20, 28)	58 (48, 67)	5.05 (4, 6)
		Total adult	41	36	28	53 (47, 57)	125 (112, 136)	10.91 (10, 12)
		Young male	4	5	4	5 (5, 5)	12 (12, 12)	1.04 (1, 1)
		Young	5	2	2	5 (5, 5)	12 (12, 12)	1.04 (1, 1)

		female						
		Total young	9	7	6	10 (8, 12)	25 (19, 29)	2.16 (2, 2)
		Total male	27	26	21	33 (30, 36)	79 (71, 86)	6.92 (6, 7)
		Total female	23	17	13	30 (25, 32)	71 (60, 76)	6.19 (5, 7)
		**Total dogs**	**50**	**43**	**34**	63 (57, 68)	150 (136, 162)	13.09 (12, 14)
KotKalan	Jalandhar	Adult Male	6	7	4	10 (7, 13)	102 (70, 130)	15.38 (11, 20)
		Adult female	5	4	3	7 (4, 8)	65 (40, 80)	9.8 (6, 12)
		Total adult	11	11	7	17 (13, 21)	170 (130, 210)	25.64 (20, 32)
		Young male	1	2	0	5 (0, 10)	50 (0, 100)	7.54 (0, 15)
		Young female	1	2	1	2 (2, 2)	20 (20, 20)	3.02 (3, 3)
		Total young	2	4	1	7 (-1, 14)	65 (-10, 140)	9.8 (-2, 21)
		Total male	7	9	4	15 (9, 21)	150 (90, 210)	22.62 (14, 32)
		Total female	6	6	4	9 (6, 11)	88 (60, 110)	13.27 (9, 17)
		**Total dogs**	**13**	**15**	**8**	24 (17, 30)	239 (170, 300)	36.03 (26, 45)
Khoba	Pathankot	Adult Male	4	3	3	4 (4, 4)	133 (133, 133)	5.05 (5, 5)
		Adult female	3	4	2	6 (3, 8)	189 (100, 267)	7.16 (4, 10)
		Total adult	7	7	5	10 (7, 12)	322 (233,	12.21 (9, 15)

							400)	
		Young male	2	1	1	2 (2, 2)	67 (67, 67)	2.53 (3, 3)
		Young female	1	1	1	1 (1, 1)	33 (33, 33)	1.26 (1, 1)
		Total young	3	2	2	3 (3, 3)	100 (100, 100)	3.79 (4, 4)
		Total male	6	4	4	6 (6, 6)	200 (200, 200)	7.58 (8, 8)
		Total female	4	5	3	7 (4, 8)	217 (133, 267)	8.21 (5, 10)
		**Total dogs**	**10**	**9**	**7**	13 (10, 15)	425 (333, 500)	16.1 (13, 19)
Gobindgarh	Fazilka	Adult Male	25	20	14	35 (29, 42)	89 (73, 105)	11.23 (9, 13)
		Adult female	15	16	12	18 (15, 20)	45 (38, 50)	5.68 (5, 6)
		Total adult	40	36	26	55 (48, 62)	138 (120, 155)	17.5 (15, 20)
		Young male	3	4	3	4 (4, 4)	10 (10, 10)	1.27 (1, 1)
		Young female	4	5	4	5 (5, 5)	13 (13, 13)	1.59 (2, 2)
		Total young	7	9	7	9 (9, 9)	23 (23, 23)	2.85 (3, 3)
		Total male	28	24	17	39 (33, 45)	98 (83, 113)	12.46 (10, 14)
		Total female	19	21	16	25 (22, 27)	62 (55, 68)	7.89 (7, 9)
		**Total dogs**	**47**	**45**	**33**	64 (57, 70)	160 (143, 175)	20.28 (18, 22)

Jethuwal	Amritsar	Adult Male	19	17	14	23 (20, 25)	100 (87, 109)	7.71 (7, 8)
		Adult female	13	11	10	14 (13, 15)	62 (57, 65)	4.78 (4, 5)
		Total adult	32	28	24	37 (34, 40)	162 (148, 174)	12.49 (11, 13)
		Young male	2	4	2	4 (4, 4)	17 (17, 17)	1.34 (1, 1)
		Young female	6	6	4	9 (6, 11)	38 (26, 48)	2.95 (2, 4)
		Total young	8	10	6	13 (9, 16)	57 (39, 70)	4.4 (3, 5)
		Total male	21	21	16	27 (24, 30)	119 (104, 130)	9.2 (8, 10)
		Total female	19	17	14	23 (20, 25)	100 (87, 109)	7.71 (7, 8)
		**Total dogs**	**40**	**38**	**30**	51 (46, 55)	220 (200, 239)	16.94 (15, 18)
Bugar	Sangrur	Adult Male	12	10	9	13 (12, 14)	190 (171, 200)	6.89 (6, 7)
		Adult female	9	10	7	13 (10, 15)	182 (143, 214)	6.61 (5, 8)
		Total adult	21	20	16	26 (23, 29)	374 (329, 414)	13.56 (12, 15)
		Young male	3	2	2	3 (3, 3)	43 (43, 43)	1.55 (2, 2)
		Young female	3	1	1	3 (3, 3)	43 (43, 43)	1.55 (2, 2)
		Total young	6	3	3	6 (6, 6)	86 (86, 86)	3.11 (3, 3)
		Total male	15	12	11	16 (15, 18)	233 (214, 257)	8.46 (8, 9)
		Total female	12	11	8	16 (13, 19)	233 (186,	8.46 (7, 10)

							271)	
		**Total dogs**	**27**	**23**	**19**	33 (29, 36)	466 (414, 514)	16.89 (15, 19)
Haraj	Mukstar	Adult Male	12	13	10	16 (13, 17)	97 (81, 106)	13.42 (11, 15)
		Adult female	11	12	8	16 (13, 19)	102 (81, 119)	14.09 (11, 16)
		Total adult	23	25	18	32 (28, 35)	199 (175, 219)	27.47 (24, 30)
		Young male	3	3	2	4 (3, 6)	27 (19, 38)	3.74 (3, 5)
		Young female	5	4	2	9 (4, 13)	56 (25, 81)	7.77 (3, 11)
		Total young	8	7	4	13 (7, 19)	84 (44, 119)	11.56 (6, 16)
		Total male	15	16	12	20 (17, 22)	125 (106, 138)	17.19 (15, 19)
		Total female	16	16	10	25 (20, 30)	158 (125, 188)	21.8 (17, 26)
		**Total dogs**	**31**	**32**	**22**	45 (39, 50)	281 (244, 313)	38.75 (34, 43)
Nabipur	Tarn Taran	Adult Male	6	7	4	10 (7, 13)	85 (58, 108)	10.21 (7, 13)
		Adult female	7	5	5	7 (7, 7)	58 (58, 58)	7.01 (7, 7)
		Total adult	13	12	9	17 (14, 20)	143 (117, 167)	17.22 (14, 20)
		Young male	6	5	4	7 (5, 9)	62 (42, 75)	7.41 (5, 9)
		Young	3	2	2	3 (3, 3)	25 (25, 25)	3 (3, 3)

		female						
		Total young	9	7	6	10 (8, 12)	87 (67, 100)	10.44 (8, 12)
		Total male	12	12	8	28 (24, 31)	231 (200, 258)	27.78 (24, 31)
		Total female	10	7	7	10 (10, 10)	83 (83, 83)	10.01 (10, 10)
		**Total dogs**	**22**	**19**	**15**	31 (25, 37)	260 (208, 308)	31.18 (25, 37)
Kheda	Mohali	Adult Male	6	8	5	10 (7, 11)	136 (100, 157)	12.31 (9, 14)
		Adult female	9	11	8	12 (11, 14)	176 (157, 200)	15.97 (14, 18)
		Total adult	15	19	13	22 (19, 24)	312 (271, 343)	28.32 (25, 31)
		Young male	1	2	1	2 (2, 2)	29 (29, 29)	2.59 (3, 3)
		Young female	1	2	1	2 (2, 2)	29 (29, 29)	2.59 (3, 3)
		Total young	2	4	2	4 (4, 4)	57 (57, 57)	5.18 (5, 5)
		Total male	7	10	6	12 (9, 13)	165 (129, 186)	14.99 (12, 17)
		Total female	10	13	9	14 (12, 16)	206 (171, 229)	18.65 (16, 21)
		**Total dogs**	**17**	**23**	**15**	26 (23, 28)	371 (329, 400)	33.68 (30, 36)
Tut	Firozpur	Adult Male	11	15	9	18 (15, 21)	114 (94, 131)	10.99 (9, 13)
		Adult	13	12	10	16 (13, 17)	97 (81, 106)	9.39 (8, 10)

		female						
		Total adult	24	27	19	34 (30, 38)	213 (188, 238)	20.53 (18, 23)
		Young male	6	4	4	6 (6, 6)	38 (38, 38)	3.62 (4, 4)
		Young female	3	4	2	5 (3, 8)	31 (19, 50)	3.02 (2, 5)
		Total young	9	8	6	12 (9, 15)	74 (56, 94)	7.16 (5, 9)
		Total male	17	19	13	25 (28, 21)	154 (175, 131)	14.92 (17, 13)
		Total female	16	16	12	21 (18, 24)	133 (113, 150)	12.82 (11, 14)
		**Total dogs**	**33**	**35**	**25**	46 (41, 51)	288 (256, 319)	27.83 (25, 31)
Sodhian	Nawanshahr	Adult Male	6	7	5	8 (7, 10)	139 (117, 167)	11.03 (9, 13)
		Adult female	4	5	4	5 (5, 5)	83 (83, 83)	6.62 (7, 7)
		Total adult	10	12	9	13 (12, 15)	222 (200, 250)	17.62 (16, 20)
		Young male	1	2	1	2 (2, 2)	33 (33, 33)	2.65 (3, 3)
		Young female	2	1	1	2 (2, 2)	33 (33, 33)	2.65 (3, 3)
		Total young	3	3	2	4 (2, 6)	72 (33, 100)	5.74 (3, 8)
		Total male	7	9	6	10 (8, 12)	174 (133, 200)	13.81 (11, 16)
		Total female	6	6	5	7 (6, 8)	120 (100, 133)	9.5 (8, 11)

		**Total dogs**	**13**	**15**	**11**	18 (15, 20)	295 (250, 333)	23.4 (20, 26)
Zeera ward 15	Firozpur	Adult Male	10	12	5	23 (14, 32)	134 (82, 188)	15.74 (10, 22)
		Adult female	13	9	6	19 (13, 24)	112 (76, 141)	13 (9, 16)
		Total adult	23	21	11	43 (31, 54)	253 (182, 318)	29.43 (21, 37)
		Young male	3	2	1	5 (2, 8)	29 (12, 47)	3.42 (1, 5)
		Young female	2	3	2	3 (3, 3)	18 (18, 18)	2.05 (2, 2)
		Total young	5	5	3	8 (4, 12)	47 (24, 71)	5.48 (3, 8)
		Total male	13	14	6	29 (18, 40)	171 (106, 235)	19.85 (12, 27)
		Total female	15	12	8	22 (16, 27)	130 (94, 159)	15.06 (11, 18)
		**Total dogs**	**28**	**26**	**14**	51 (39, 63)	301 (229, 371)	34.91 (27, 43)
Zeera ward 16	Firozpur	Adult Male	19	23	11	39 (29, 49)	30 (22, 38)	22.52 (17, 28)
		Adult female	15	17	12	21 (18, 24)	16 (14, 19)	12.12 (10, 14)
		Total adult	34	40	23	59 (50, 67)	46 (39, 52)	34.06 (29, 39)
		Young male	6	5	3	10 (6, 13)	7 (5, 10)	5.77 (3, 8)
		Young	5	4	3	7 (4, 8)	5 (3, 6)	4.04 (2, 5)

		female						
		Total young	11	9	6	16 (11, 21)	13 (9, 16)	9.24 (6, 12)
		Total male	25	28	14	49 (38, 60)	38 (29, 47)	28.29 (22, 35)
		Total female	20	21	15	28 (24, 31)	22 (19, 24)	16.17 (14, 18)
		**Total dogs**	**45**	**49**	**29**	76 (65, 86)	59 (50, 67)	43.88 (38, 50)
Ludhiana ward 62	Ludhiana	Adult Male	31	26	17	47 (38, 55)	100 (81, 117)	7.33 (6, 9)
		Adult female	20	17	12	28 (23, 33)	60 (49, 70)	4.38 (4, 5)
		Total adult	51	43	29	75 (65, 85)	160 (138, 181)	11.73 (10, 13)
		Young male	9	7	4	15 (9, 21)	32 (19, 45)	2.34 (1, 3)
		Young female	7	9	3	19 (9, 29)	40 (19, 62)	2.96 (1, 5)
		Total young	16	16	7	35 (20, 49)	75 (43, 104)	5.48 (3, 8)
		Total male	40	33	21	62 (51, 73)	133 (109, 155)	9.72 (8, 11)
		Total female	27	26	15	46 (36, 56)	98 (77, 119)	7.21 (6, 9)
		**Total dogs**	**67**	**59**	**36**	109 (94, 124)	232 (200, 264)	17.03 (15, 19)
Ludhiana ward 63	Ludhiana	Adult Male	38	33	20	62 (51, 73)	51 (42, 60)	3.87 (3, 5)
		Adult	28	30	16	52 (41, 62)	43 (34, 51)	3.23 (3, 4)

		female						
		Total adult	66	63	36	115 (98, 131)	95 (81, 108)	7.15 (6, 8)
		Young male	11	12	8	16 (13, 19)	14 (11, 16)	1.02 (1, 1)
		Young female	14	13	9	20 (16, 24)	17 (13, 20)	1.24 (1, 1)
		Total young	25	25	17	37 (31, 42)	30 (26, 35)	2.28 (2, 3)
		Total male	49	45	28	78 (67, 90)	65 (55, 74)	4.87 (4, 6)
		Total female	42	43	25	72 (60, 83)	59 (50, 69)	4.47 (4, 5)
		**Total dogs**	**91**	**88**	**53**	151 (13, 167)	124 (11, 138)	9.38 (1, 10)
Bhagta Bhaika ward 8	Bathinda	Adult Male	4	6	2	11 (5, 16)	213 (100, 320)	9.39 (4, 14)
		Adult female	5	3	2	7 (4, 10)	140 (80, 200)	6.16 (4, 9)
		Total adult	9	9	4	19 (10, 27)	380 (200, 540)	16.73 (9, 24)
		Young male	1	2	1	2 (2, 2)	40 (40, 40)	1.76 (2, 2)
		Young female	1	1	0	3 (0, 6)	60 (0, 120)	2.64 (0, 5)
		Total young	2	3	1	5 (0, 11)	100 (0, 220)	4.4 (0, 10)
		Total male	5	8	3	13 (7, 18)	250 (140, 360)	11 (6, 16)
		Total female	6	4	2	11 (5, 16)	213 (100, 320)	9.39 (4, 14)
		**Total dogs**	**11**	**12**	**5**	25 (14, 35)	500 (280, 700)	22.01 (12, 31)
Bhagta Bhaika	Bathinda	Adult Male	9	8	5	14 (9, 18)	100 (64, 129)	14.52 (9, 19)

ward 1								
		Adult female	5	4	2	9 (4, 13)	64 (29, 93)	9.34 (4, 13)
		Total adult	14	12	7	23 (16, 30)	167 (114, 214)	24.25 (17, 31)
		Young male	2	3	1	5 (2, 8)	36 (14, 57)	5.19 (2, 8)
		Young female	4	5	3	7 (4, 8)	46 (29, 57)	6.74 (4, 8)
		Total young	6	8	4	12 (7, 16)	83 (50, 114)	12.03 (7, 17)
		Total male	11	11	6	20 (13, 25)	140 (93, 179)	20.3 (13, 26)
		Total female	9	9	5	16 (10, 20)	112 (71, 143)	16.26 (10, 21)
		**Total dogs**	**20**	**20**	**11**	36 (27, 44)	255 (193, 314)	37.09 (28, 46)
Balachaur ward 4	Nawanshahr	Adult Male	19	25	10	46 (32, 60)	231 (160, 300)	43.41 (30, 56)
		Adult female	23	20	14	33 (27, 38)	163 (135, 190)	30.58 (25, 36)
		Total adult	42	45	33	78 (64, 91)	391 (320, 455)	73.28 (60, 85)
		Young male	7	4	3	9 (6, 12)	45 (30, 60)	8.44 (6, 11)
		Young female	3	4	3	4 (4, 4)	20 (20, 20)	3.75 (4, 4)
		Total young	10	8	6	13 (9, 16)	66 (45, 80)	12.33 (8, 15)
		Total male	22	29	19	57 (41, 71)	284 (205, 355)	53.34 (38, 67)
		Total female	30	24	20	37 (31, 42)	183 (155,	34.24 (29,

							210)	39)
		**Total dogs**	**52**	**53**	**39**	91 (77, 105)	457 (385, 525)	85.67 (72, 98)
Balachaur ward 5	Nawanshahr	Adult Male	5	6	3	10 (6, 13)	190 (120, 260)	8.36 (5, 11)
		Adult female	3	2	1	5 (2, 8)	100 (40, 160)	4.4 (2, 7)
		Total adult	8	8	4	15 (9, 21)	304 (180, 420)	13.38 (8, 18)
		Young male	0	2	0	2 (2, 2)	40 (40, 40)	1.76 (2, 2)
		Young female	2	3	2	3 (3, 3)	60 (60, 60)	2.64 (3, 3)
		Total young	2	5	2	5 (5, 5)	100 (100, 100)	4.4 (4, 4)
		Total male	5	8	3	13 (7, 18)	250 (140, 360)	11 (6, 16)
		Total female	5	5	3	8 (5, 11)	160 (100, 220)	7.04 (4, 10)
		**Total dogs**	**10**	**13**	**6**	21 (14, 28)	420 (280, 560)	18.49 (12, 25)
Bathinda ward 9	Bathinda	Adult Male	15	12	6	29 (18, 39)	78 (49, 105)	7.77 (5, 11)
		Adult female	9	8	5	14 (9, 18)	38 (24, 49)	3.79 (2, 5)
		Total adult	24	20	11	43 (31, 54)	116 (84, 146)	11.57 (8, 15)
		Young male	5	5	3	8 (5, 11)	22 (14, 30)	2.17 (1, 3)

		Young female	3	5	2	7 (4, 10)	19 (11, 27)	1.89 (1, 3)
		Total young	8	10	5	16 (9, 21)	42 (24, 57)	4.19 (2, 6)
		Total male	20	17	9	37 (25, 48)	99 (68, 130)	9.96 (7, 13)
		Total female	12	13	7	22 (15, 28)	59 (41, 76)	5.89 (4, 8)
		**Total dogs**	**32**	**30**	**16**	59 (46, 72)	160 (124, 195)	16.02 (12, 19)
Bathinda ward 11	Bathinda	Adult Male	14	18	8	31 (21, 40)	63 (43, 82)	7.02 (5, 9)
		Adult female	16	19	10	30 (22, 37)	61 (45, 76)	6.84 (5, 8)
		Total adult	30	37	18	61 (49, 73)	124 (100, 149)	13.96 (11, 17)
		Young male	7	4	3	9 (6, 12)	18 (12, 24)	2.06 (1, 3)
		Young female	4	5	3	7 (4, 8)	13 (8, 16)	1.49 (1, 2)
		Total young	11	9	6	16 (11, 21)	33 (22, 43)	3.69 (3, 5)
		Total male	21	22	11	41 (30, 52)	84 (61, 106)	9.42 (7, 12)
		Total female	20	24	13	37 (29, 44)	74 (59, 90)	8.35 (7, 10)
		**Total dogs**	**41**	**46**	**24**	78 (64, 92)	159 (131, 188)	17.84 (15, 21)

Table S3: Total number of stray dogs estimated in different village(s)/ward(s) in Punjab state of India (using *SuperDuplicates method*).

						Incidence data	
**Name of the village/ward**	**District**	**Type of dog**	**n1 (day 1)**	**n2 (day 2)**	**M (re-sighted)**	**Estimate ± SE (95% CI)**	**Undetected % (N)**
Galib Ran Singh	Ludhiana	Adult Male	13	11	10	28.90 ± 4.10 (25.18-44.43)	16.96 (4.90)
		Adult female	9	10	6	26.04 ± 10.18 (19.88-75.48)	27.04 (7.04)
		Total adult	22	21	16	54.39 ± 6.36 (47.1-74.62)	20.94 (11.39)
		Young male	3	4	3	8.33 ± 2.75 (7.11-23.64)	16 (1.33)
		Young female	2	1	1	4 ± 1.42 (3.13-10.86)	25 (1)
		Total young	5	5	4	12.25 ± 3.92 (10.22-32.77)	18.37 (2.25)
		Total male	16	15	13	37.23 ± 4.45 (32.77-52.91)	16.74 (6.23)
		Total female	11	11	7	30.04 ± 7012 (23.81-57.71)	26.75 (8.04)
		**Total dogs**	**27**	**26**	**20**	66.61 ± 7.08 (58.22-88.5	20.44(13.61)
Pipli	Faridkot	Adult Male	18	21	12	54.19 ± 8.5 (44.46-81.25)	28.03 (15.19)

		Adult female	17	18	13	44.31 ± 6.68 (37.63-67.94)	21.01 (9.31)
		Total adult	35	39	25	132.02 ± 24.42 (100.29- 202.05)	43.95 (58.02)
		Young male	5	4	2	15.12 ± 8.71 (9.78 - 57.15)	40.5 (6.12)
		Young female	3	5	2	12.5 ± 6.11 (8.61 - 41.34)	36 (4.5)
		Total young	8	9	4	27.56 ± 15.01 (18.35-99.91)	38.32 (10.56)
		Total male	23	25	14	68.64 ± 11.5 (55.45-105.2)	30.07 (20.64)
		Total female	20	23	15	56.07 ± 8.06 (47.29-82.78)	23.31 (13.07)
		**Total dogs**	**43**	**48**	**29**	124.14 ± 13.03 (106.76-160.67)	26.69 (33.14)
Jodhpur	Barnala	Adult Male	21	19	14	52.07 ± 7.6 (43.89-77.47)	23.18 (12.07)
		Adult female	17	20	15	45.07 ± 5.06 (39.61-61.97)	17.9 (8.07)
		Total adult	38	39	29	96.86 ± 8.05 (86.25-119.64)	20.51 (19.86)
		Young male	3	4	2	10.12 ± 4.62 (7.38-32.8)	30.86 (3.12)
		Young female	6	5	4	14.06 ± 5.66 (11.28-44.38)	21.78 (3.06)
		Total young	9	9	6	24 ± 8.38 (18.78 -	25 (6)

						64.12)	
		Total male	24	23	16	62.02 ± 7.78 (52.77-86.06)	24.21 (15.02)
		Total female	23	25	19	59.07 ± 5.99(52.09-77.91)	18.73 (11.07)
		**Total dogs**	**47**	**48**	**35**	120.71 ± 9.58 (107.69-147.12)	21.3 (25.71)
Rauli	Hoshiarpur	Adult Male	5	4	3	12 ± 5.1 (9.31 -38.44)	25 (3)
		Adult female	6	5	4	14.06 ± 5.66 (11.28-44.38)	21.78 (3.06)
		Total adult	11	9	7	26.04 ± 6.11 (21.16-51.29)	23.18 (6.04)
		Young male	1	2	1	4 ± 1.42 (3.13-10.86)	25 (1)
		Young female	2	2	1	6.25 ± 2.42 (4.4 -16.52)	36 (2.25)
		Total young	3	4	2	10.12 ± 4.62 (7.38-32.8)	30.86 (3.12)
		Total male	6	6	4	16 ± 7.23 (12.38-54.43)	25 (4)
		Total female	8	7	5	20 ± 6.31 (15.74-48.89)	25 (5)
		**Total dogs**	**14**	**13**	**9**	36 ± 1 7.13 (29.29-62.35)	25 (9)
Mangewala	Moga	Adult Male	16	18	13	42.48 ± 5.97(36.44-63.42)	19.96 (8.48)
		Adult female	15	12	10	34.23 ± 6.3 (28.65-	21.11 (7.23)

						58.54)	
		Total adult	31	30	23	76.7 ± 7.32 (67.58-98.47)	20.46 (15.7)
		Young male	4	3	3	8.33 ± 2.75 (7.11-23.64)	16 (1.33)
		Young female	3	4	2	10.12 ± 4.62 (7.38-32.8)	30.86 (3.12)
		Total young	7	7	5	18.05 ± 6.45 (14.45-5066)	22.44 (4.05)
		Total male	20	21	16	50.77 ± 7.47 (43.58-77.94)	19.24 (9.77)
		Total female	18	16	12	44.08 ± 7.47 (36.76-70.88)	22.87 (10.08)
		**Total dogs**	**38**	**37**	**28**	94.72 ± 8.51 (83.77-119.34)	20.82 (19.72)
Karahal Kalan	Kapurthala	Adult Male	8	6	4	20.25 ± 12.08 (14.54-86.02)	30.86 (6.25)
		Adult female	4	5	4	10.56 ± 2.88 (9.14-25.95)	14.79 (1.56)
		Total adult	12	11	8	30.03 ± 6.79 (24.43-57.49)	23.41 (7.03)
		Young male	2	3	1	9 ± 3.34 (5.96-21.66)	44.44 (4)
		Young female	3	4	3	8.33 ± 2.75 (7.11-23.64)	16 (1.33)
		Total young	5	7	4	16 ± 7.23 (12.38-54.43)	25 (4)
		Total male	10	9	5	28.8 ± 12.95 (20.37-	34.03 (9.8)

						89.26)	
		Total female	7	9	7	18.89 ± 3.8 (16.41-36.59)	15.31 (2.89)
		**Total dogs**	**17**	**18**	**12**	46.02 ± 7.42 (38.33-71.51)	23.95 (11.02)
Devgarh	Patiala	Adult Male	13	10	7	32.14 ± 8.34 (24.99-65.03)	28.44 (9.14)
		Adult female	7	6	5	16.2 ± 7.14 (13.23-56.98)	19.75 (3.2)
		Total adult	20	16	12	48 ± 7.84 (39.73-74.59)	25 (12)
		Young male	2	2	2	4.5 ± 0.84 (4.05-8.84)	11.11 (0.5)
		Young female	4	3	3	8.33 ± 2.75 (7.11-23.64)	16 (1.33)
		Total young	6	5	5	12.8 ± 3.22 (11.17-29.86)	14.06 (1.8)
		Total male	15	12	9	36 ± 1 7.13 (29.29-62.35)	25 (9)
		Total female	11	9	8	24.5 ± 4.25 (20.94-41.5)	18.37 (4.5)
		**Total dogs**	**26**	**21**	**17**	60.24 ± 7.2 (51.88-82.9)	21.97 (13.24)
Mander	Mansa	Adult Male	21	18	17	46.12 ± 4.69 (41.19-62.11)	15.43 (7.12)
		Adult female	16	14	12	36.75 ± 5.18 (31.78-55.64)	18.37 (6.75)

		Total adult	37	32	29	82.79 ± 7.28 (74.22-105.42)	16.66 (13.79)
		Young male	3	4	3	8.33 ± 2.75 (7.11-23.64)	16 (1.33)
		Young female	3	3	2	8 ± 3.69 (6.18-27.74)	25 (2)
		Total young	6	7	5	16.2 ± 7.14 (13.23-56.98)	19.75 (3.2)
		Total male	24	22	20	54.45 ± 4.84 (48.97-70.01)	15.52 (8.45)
		Total female	19	17	14	44.64 ± 7.01 (38.15-70.81)	19.36 (8.64)
		**Total dogs**	**43**	**39**	**34**	98.94 ± 6.99 (89.79-118.84)	17.12 (16.94)
Rali Ali Kalan	Gurdaspur	Adult Male	6	7	3	21.33 ± 10.28 (14.27-67.87)	39.06 (8.33)
		Adult female	4	5	2	15.12 ± 8.71 (9.78 - 57.15)	40.5 (6.12)
		Total adult	10	12	5	36.45 ± 17.33 (24.27-113.96)	39.64 (14.45)
		Young male	6	3	2	15.12 ± 8.71 (9.78 - 57.15)	40.5 (6.12)
		Young female	2	2	2	4.5 ± 0.84 (4.05-8.84)	11.11 (0.5)
		Total young	8	5	4	18.06 ± 7.81 (13.58-57.02)	28.03 (5.06)
		Total male	12	10	5	36.45 ± 17.33 (24.27-113.96)	39.64 (14.45)
		Total female	6	7	4	18.06 ± 7.81 (13.58-	28.03 (5.06)

						57.02)	
		**Total dogs**	**18**	**17**	**9**	53.78 ± 15.03 (39.73-109.6)	34.92 (18.78)
Mahdian	Fatehgarh Sahib	Adult Male	9	7	6	20.17 ± 6.43 (16.48-52.25)	20.66 (4.17)
		Adult female	4	6	3	14.08 ± 5.71 (10.53-41.4)	28.99 (4.08)
		Total adult	13	13	9	34.03 ± 8.16 (27.54-67.84)	23.59 (8.03)
		Young male	1	2	0		
		Young female	3	4	3	8.33 ± 2.75 (7.11-23.64)	16 (1.33)
		Total young	4	6	3	14.08 ± 5.71 (10.53-41.4)	28.99 (4.08)
		Total male	10	9	6	26.04 ± 10.18 (19.88-75.48)	27.04 (7.04)
		Total female	7	10	6	22.04 ± 5.65 (17.86-46.55)	22.87 (5.04)
		**Total dogs**	**17**	**19**	**12**	48 ± 8.71 (39.35-78.97)	25 (12)
Rangian	Rupnagar (Ropar)	Adult Male	11	12	8	30.03 ± 6.79 (24.43-57.49)	23.41 (7.03)
		Adult female	6	7	5	16.2 ± 7.14 (13.23-56.98)	19.75 (3.2)
		Total adult	17	19	13	46.17 ± 7.23 (38.91-71.61)	22.03 (10.17)

		Young male	1	1	1	2.25 ± 0.21 (2.06-3.07)	11.11 (0.25)
		Young female	3	3	2	8 ± 3.69 (6.18-27.74)	25 (2)
		Total young	4	4	3	10.08 ± 4.38 (8.16-34.6)	20.66 (2.08)
		Total male	12	13	9	32.11 ± 6.81 (26.46-59.56)	22.15 (7.11)
		Total female	9	10	7	24.14 ± 4.46 (20.18-41.33)	21.3(5.14)
		**Total dogs**	**21**	**23**	**16**	56.25 ± 6.75 (48.47-77.6)	21.78 (12.25)
Gobindpura	Bathinda	Adult Male	23	21	17	54.72 ± 7.79 (46.99-82.42)	19.59 (10.72)
		Adult female	18	15	11	44 ± 7.2 (36.41-68.43)	25 (11)
		Total adult	41	36	28	98.44 ± 9.89 (86.07-127.69)	21.78 (21.44)
		Young male	4	5	4	10.56 ± 2.88 (9.14-25.95)	14.79 (1.56)
		Young female	5	2	2	10.12 ± 4.62 (7.38-32.8)	30.86 (3.12)
		Total young	9	7	6	20.17 ± 6.43 (16.48-52.25)	20.66 (4.17)
		Total male	27	26	21	65.19 ± 6.7 (57.45-86.37)	18.7 (12.19)
		Total female	23	17	13	54.02 ± 7.65 (45.15-78.15)	25.95 (14.02)
		**Total dogs**	**50**	**43**	**34**	118.6 ± 10.29 (104.99-147.65)	21.58 (25.6)

KotKalan	Jalandhar	Adult Male	6	7	4	18.06 ± 7.81 (13.58-57.02)	28.03 (5.06)
		Adult female	5	4	3	12 ± 5.1 (9.31 -38.44)	25 (3)
		Total adult	11	11	7	30.04 ± 7012 (23.81-57.71)	26.75 (8.04)
		Young male	1	2	0		
		Young female	1	2	1	4 ± 1.42 (3.13-10.86)	25 (1)
		Total young	2	4	1	12.25 ± 5.54 (7.41-33.8)	51.02 (6.25)
		Total male	7	9	4	25 ± 12.14 (17.22-82.22)	36 (9)
		Total female	6	6	4	16 ± 7.23 (12.38-54.43)	25 (4)
		**Total dogs**	**13**	**15**	**8**	40.5 ± 18.47 (29.52-130.99)	30.86 (12.5)
Khoba	Pathankot	Adult Male	4	3	3	8.33 ± 2.75 (7.11-23.64)	16 (1.33)
		Adult female	3	4	2	10.12 ± 4.62 (7.38-32.8)	30.86 (3.12)
		Total adult	7	7	5	18.05 ± 6.45 (14.45-5066)	22.44 (4.05)
		Young male	2	1	1	4 ± 1.42 (3.13-10.86)	25 (1)
		Young female	1	1	1	2.25 ± 0.21 (2.06-3.07)	11.11 (0.25)
		Total young	3	2	2	6.12 ± 1.95 (5.11-16.34)	18.37 (1.12)
		Total male	6	4	4	12.25 ± 3.92 (10.22-	18.37 (2.25)

						32.77)	
		Total female	4	5	3	12 ± 5.1 (9.31 -38.44)	25 (3)
		**Total dogs**	**10**	**9**	**7**	24.14 ± 4.46 (20.18-41.33)	21.3(5.14)
Gobindgarh	Fazilka	Adult Male	25	20	14	62.16 ± 11.06 (50.41-99.46)	27.61 (17.16)
		Adult female	15	16	12	38.52 ± 5.28 (33.17-57.01)	19.52 (7.52)
		Total adult	40	36	26	100.04 ± 10.21 (86.82-129.41)	24.03 (24.04)
		Young male	3	4	3	8.33 ± 2.75 (7.11-23.64)	16 (1.33)
		Young female	4	5	4	10.56 ± 2.88 (9.14-25.95)	14.79 (1.56)
		Total young	7	9	7	18.89 ± 3.8 (16.41-36.59)	15.31 (2.89)
		Total male	28	24	17	70.01 ± 10.83 (58.07-105.51)	25.73 (18.01)
		Total female	19	21	16	49 ± 6 (42.74-69.53)	18.37 (9)
		**Total dogs**	**47**	**45**	**33**	118.37 ± 11.02 (104.01-149.91)	22.28 (26.37)
Jethuwal	Amritsar	Adult Male	19	17	14	44.64 ± 7.01 (38.15-70.81)	19.36 (8.64)
		Adult female	13	11	10	28.90 ± 4.10 (25.18-44.43)	16.96 (4.90)
		Total adult	32	28	24	73.5 ± 7.02 (65.18-	18.37 (13.5)

						95.21)	
		Young male	2	4	2	8 ± 3.69 (6.18-27.74)	25 (2)
		Young female	6	6	4	16 ± 7.23 (12.38-54.43)	25 (4)
		Total young	8	10	6	24 ± 8.38 (18.78 -64.12)	25 (6)
		Total male	21	21	16	52.56 ± 7.32 (45.09-78.05)	20.1 (10.56)
		Total female	19	17	14	44.64 ± 7.01 (38.15-70.81)	19.36 (8.64)
		**Total dogs**	**40**	**38**	**30**	97.2 ± 7.11 (87.51-116.76)	19.75 (19.2)
Bugar	Sangrur	Adult Male	12	10	9	26.69 ± 4.43 (22.98-44.44)	17.59 (4.69)
		Adult female	9	10	7	24.14 ± 4.46 (20.18-41.33)	21.3(5.14)
		Total adult	21	20	16	50.77 ± 5.94 (44.25-70.35)	19.24 (9.77)
		Young male	3	2	2	6.12 ± 1.95 (5.11-16.34)	18.37 (1.12)
		Young female	3	1	1	6.25 ± 2.42 (4.4 -16.52)	36 (2.25)
		Total young	6	3	3	12 ± 5.1 (9.31 -38.44)	25 (3)
		Total male	15	12	11	32.82 ± 4.58 (28.49-49.7)	17.73 (5.82)
		Total female	12	11	8	30.03 ± 6.79 (24.43-57.49)	23.41 (7.03)

		**Total dogs**	**27**	**23**	**19**	62.64 ± 6.68 (54.78-83.45)	20.18 (12.64)
Haraj	Mukstar	Adult Male	12	13	10	30.62 ± 6.09 (26-56.62)	18.37 (5.62)
		Adult female	11	12	8	30.03 ± 6.79 (24.43-57.49)	23.41 (7.03)
		Total adult	23	25	18	60.5 ± 7.84 (52.04-86.67)	20.66 (12.5)
		Young male	3	3	2	8 ± 3.69 (6.18-27.74)	25 (2)
		Young female	5	4	2	15.12 ± 8.71 (9.78 - 57.15)	40.5 (6.12)
		Total young	8	7	4	22.56 ± 8.92 (16.21-62.17)	33.52 (7.56)
		Total male	15	16	12	38.52 ± 5.28 (33.17-57.01)	19.52 (7.52)
		Total female	16	16	10	44.1 ± 8.85 (35.35-75.64)	27.44 (12.1)
		**Total dogs**	**31**	**32**	**22**	82.1 ± 10.16 (70.18-113.83)	23.27 (19.1)
Nabipur	Tarn Taran	Adult Male	6	7	4	18.06 ± 7.81 (13.58-57.02)	28.03 (5.06)
		Adult female	7	5	5	14.45 ± 4.71 (12.21-40.08)	16.96 (2.45)
		Total adult	13	12	9	32.11 ± 6.81 (26.46-59.56)	22.15 (7.11)
		Young male	6	5	4	12.8 ± 3.22 (11.17-	14.06 (1.8)

						29.86)	
		Young female	3	2	2	6.12 ± 1.95 (5.11-16.34)	18.37 (1.12)
		Total young	9	7	6	20.17 ± 6.43 (16.48-52.25)	20.66 (4.17)
		Total male	12	12	8	32 ± 7.82 (25.61-63.8)	25 (8)
		Total female	10	7	7	20.57 ± 4.22 (17.57-39.29)	17.36 (3.57)
		**Total dogs**	**22**	**19**	**15**	52.27 ± 6.85 (44.76-74.8)	21.56 (11.27)
Kheda	Mohali	Adult Male	6	8	5	18.05 ± 6.45 (14.45-5066)	22.44 (4.05)
		Adult female	9	11	8	24.5 ± 4.25 (20.94-41.5)	18.37 (4.5)
		Total adult	15	19	13	42.48 ± 5.97(36.44-63.42)	19.96 (8.48)
		Young male	1	2	1	4 ± 1.42 (3.13-10.86)	25 (1)
		Young female	1	2	1	4 ± 1.42 (3.13-10.86)	25 (1)
		Total young	2	4	2	8 ± 3.69 (6.18-27.74)	25 (2)
		Total male	7	10	6	22.04 ± 5.65 (17.86-46.55)	22.87 (5.04)
		Total female	10	13	9	28.44 ± 5.02 (24.17-48.32)	19.14 (5.44)
		**Total dogs**	**17**	**23**	**15**	50.42 ± 6.87(43.21-73.84)	20.66 (10.42)
Tut	Firozpur	Adult Male	11	15	9	34.03 ± 8.16 (27.54-	23.59 (8.03)

						67.84)	
		Adult female	13	12	10	30.62 ± 6.09 (26-56.62)	18.37 (5.62)
		Total adult	24	27	19	64.47 ± 7.96 (55.61-90.39)	20.9 (13.47)
		Young male	6	4	4	12.25 ± 3.92 (10.22-32.77)	18.37 (2.25)
		Young female	3	4	2	10.12 ± 4.62 (7.38-32.8)	30.86 (3.12)
		Total young	9	8	6	22.04 ± 5.65 (17.86-46.55)	22.87 (5.04)
		Total male	17	19	13	46.17 ± 6.63 (39.17-68.65)	22.03 (10.17)
		Total female	16	16	12	40.33 ± 6.04 (34.33-61.77)	20.66 (8.33)
		**Total dogs**	**33**	**35**	**25**	86.49 ± 8.43 (75.89-111.33)	21.38 (18.49)
Sodhian	Nawanshahr	Adult Male	6	7	5	16.2 ± 7.14 (13.23-56.98)	19.75 (3.2)
		Adult female	4	5	4	10.56 ± 2.88 (9.14-25.95)	14.79 (1.56)
		Total adult	10	12	9	26.69 ± 4.43 (22.98-44.44)	17.59 (4.69)
		Young male	1	2	1	4 ± 1.42 (3.13-10.86)	25 (1)
		Young female	2	1	1	4 ± 1.42 (3.13-10.86)	25 (1)
		Total young	3	3	2	8 ± 3.69 (6.18-27.74)	25 (2)
		Total male	7	9	6	20.17 ± 6.43 (16.48-	20.66 (4.17)

							52.25)	
			Total female	6	6	5	14.45 ± 4.71 (12.21-40.08)	16.96 (2.45)
			**Total dogs**	**13**	**15**	**11**	34.57 ± 5.43 (29.59-55.05)	19 (6.57)
Zeera	ward 15	Firozpur	Adult Male	10	12	5	36.45 ± 17.33 (24.27-113.96)	39.64 (14.45)
			Adult female	13	9	6	32.67 ± 13.13 (23.62-92.08)	32.65 (10.67)
			Total adult	23	21	11	68.75 ± 14.66 (52.45-116.53)	36 (24.75)
			Young male	3	2	1	9 ± 3.34 (5.96-21.66)	44.44 (4)
			Young female	2	3	2	6.12 ± 1.95 (5.11-16.34)	18.37 (1.12)
			Total young	5	5	3	14.08 ± 5.71 (10.53-41.4)	28.99 (4.08)
			Total male	13	14	6	45.38 ± 17.2 (30.88-114)	40.5 (18.38)
			Total female	15	12	8	55.26 ± 9.52 (35.31-123.13)	51.14 (28.26)
			**Total dogs**	**28**	**26**	**14**	82.57 ± 13.52 (65.84-122.97)	34.6 (28.57)
Zeera	ward 16	Firozpur	Adult Male	19	23	11	63.84 ± 11.59 (50.23-99.97)	34.21 (21.84)
			Adult female	15	17	12	40.33 ± 6.04 (34.33-61.77)	20.66 (8.33)

		Total adult	34	40	23	102.27 ± 11.43 (87.19-134.6)	27.64 (28.27)
		Young male	6	5	3	16.33 ± 9.57 (11.51-67.05)	32.65 (5.33)
		Young female	5	4	3	12 ± 5.1 (9.31 -38.44)	25 (3)
		Total young	11	9	6	28.17 ± 11.14 (21.09-80.93)	28.99 (8.17)
		Total male	25	28	14	80.16 ± 14.28 (63.32-124.51)	31.23 (24.07)
		Total female	20	21	15	52.27 ± 6.85 (44.76-74.8)	21.56 (11.27)
		**Total dogs**	**45**	**49**	**29**	130.42 ± 13.84 (111.73-168.82)	27.93 (36.42)
Ludhiana ward 62	Ludhiana	Adult Male	31	26	17	80.53 ± 11.19 (66.71-114.03)	29.22 (23.53)
		Adult female	20	17	12	50.02 ± 8.62 (41-79.43)	26.03 (13.02)
		Total adult	51	43	29	130.42 ± 13.84 (111.73-168.82)	27.93 (36.42)
		Young male	9	7	4	25 ± 12.14 (17.22-82.22)	36 (9)
		Young female	7	9	3	30.08 ± 13.14 (18.99-82.4)	46.81 (14.08)
		Total young	16	16	7	54.32 ± 24.37 (35.93-158.72)	41.09 (22.32)
		Total male	40	33	21	105.19 ± 13.61 (87.54-144.28)	30.6 (32.19)

		Total female	27	26	15	77.07 13.12 (61.86-118.4)	31.23 (24.07)
		**Total dogs**	**67**	**59**	**36**	182.25 ± 18.58 (155.94-231.69)	30.86 (56.25)
Ludhiana ward 63	Ludhiana	Adult Male	38	33	20	103.51 ± 15.58 (84.34-150.27)	31.41 (32.51)
		Adult female	28	30	16	85.56 ± 13.99 (68.78-128.45)	32.21 (27.56)
		Total adult	66	63	36	189.06 ± 21.44 (159.47-247.39)	31.77 (60.06)
		Young male	11	12	8	30.03 ± 6.79 (24.43-57.49)	23.41 (7.03)
		Young female	14	13	9	36 ± 17.13 (29.29-62.35)	25 (9)
		Total young	25	25	17	66.01 8.43 (56.07-92.22)	24.26 (16.01)
		Total male	49	45	28	132.89 ± 14.66 (113.04-173.45)	29.27 (38.89)
		Total female	42	43	25	121 ± 14.45 (101.88-161.8)	29.75 (36)
		**Total dogs**	**91**	**88**	**53**	253.89 ± 20.06 (223.7-304.46)	29.5 (74.89)
Bhagta Bhaika ward 8	Bathinda	Adult Male	4	6	2	18 ± 9.13 (11.34-57.9)	44.44 (8)
		Adult female	5	3	2	12.5 ± 6.11 (8.61 - 41.34)	36 (4.5)

		Total adult	9	9	4	30.25 ± 14.76 (19.91-96.41)	40.5 (12.25)
		Young male	1	2	1	4 ± 1.42 (3.13-10.86)	25 (1)
		Young female	1	1	0		
		Total young	2	3	1	9 ± 3.34 (5.96-21.66)	44.44 (4)
		Total male	5	8	3	21.33 ± 10.28 (14.27-67.87)	39.06 (8.33)
		Total female	6	4	2	18 ± 9.13 (11.34-57.9)	44.44 (8)
		**Total dogs**	**11**	**12**	**5**	39.2 ± 21.63 (25.23-140.66)	41.33 (16.2)
Bhagta Bhaika ward 1	Bathinda	Adult Male	9	8	5	24.2 ± 9.63 (69.43-17.99)	29.75 (7.2)
		Adult female	5	4	2	15.12 ± 8.71 (9.78 -57.15)	40.5 (6.12)
		Total adult	14	12	7	38.89 ± 11.97 (28.75-86.47)	33.15 (12.89)
		Young male	2	3	1	9 ± 3.34 (5.96-21.66)	44.44 (4)
		Young female	4	5	3	12 ± 5.1 (9.31 -38.44)	25 (3)
		Total young	6	8	4	20.25 ± 12.08 (14.54-86.02)	30.86 (6.25)
		Total male	11	11	6	32.67 ± 13.13 (23.62-92.08)	32.65 (10.67)
		Total female	9	9	5	26.45 ± 8.58 (19.62-61.99)	31.95 (8.45)
		**Total dogs**	**20**	**20**	**11**	59.11 ± 10.86 (46.78-93.89)	32.33 (19.11)

Balachaur ward 4	Nawanshahr	Adult Male	19	25	10	72.9 ± 15.9 (54.55-123.18)	39.64 (28.9)
		Adult female	23	20	14	58.02 ±9.28 (47.92-88.8)	25.88 (15.02)
		Total adult	42	45	24	128.34 ± 17.52 (105.65-178.68)	32.21 (41.34)
		Young male	7	4	3	16.33 ± 9.57 (11.51-67.05)	32.65 (5.33)
		Young female	3	4	3	8.33 ± 2.75 (7.11-23.64)	16 (1.33)
		Total young	10	8	6	24 ± 8.38 (18.78 - 64.12)	25 (6)
		Total male	26	29	13	88.92 ± 18 (67.78-145.03)	38.15 (33.92)
		Total female	26	24	17	66.01 ± 9.65 (55.38-97.68)	24.26 (16.01)
		**Total dogs**	**52**	**53**	**30**	151.88 ± 15.77 (129.67-194.06)	30.86 (46.88)
Balachaur ward 5	Nawanshahr	Adult Male	5	6	3	16.33 ± 9.57 (11.51-67.05)	32.65 (5.33)
		Adult female	3	2	1	9 ± 3.34 (5.96-21.66)	44.44 (4)
		Total adult	8	8	4	25 ± 12.14 (17.22-82.22)	36 (9)
		Young male	0	2	0		
		Young female	2	3	2	9 ± 3.34 (5.96-21.66)	44.44 (4)
		Total young	2	5	2	10.12 ± 4.62 (7.38-32.8)	30.86 (3.12)

		Total male	5	8	3	21.33 ± 10.28 (14.27-67.87)	39.06 (8.33)
		Total female	5	5	3	14.08 ± 5.71 (10.53-41.4)	28.99 (4.08)
		**Total dogs**	**10**	**13**	**6**	35.04 ± 13.89 (24.98-96.08)	34.36 (12.04)
Bathinda ward 9	Bathinda	Adult Male	15	12	6	45.38 ± 18.12 (30.65-119.42)	40.5 (18.38)
		Adult female	9	8	5	24.2 ± 9.63 (69.43-17.99)	29.75 (7.2)
		Total adult	24	20	11	68.75 ± 14.66 (52.45-116.53)	36 (24.75)
		Young male	5	5	3	14.08 ± 5.71 (10.53-41.4)	28.99 (4.08)
		Young female	3	5	2	12.5 ± 6.11 (8.61 - 41.34)	36 (4.5)
		Total young	8	10	5	26.45 ± 8.58 (19.62-61.99)	31.95 (8.45)
		Total male	20	17	9	58.78 ± 16.84 (42.69-120.32)	37.05 (21.78)
		Total female	12	13	7	36.57 ± 10.8 (27.45-79.6)	31.64 (11.57)
		**Total dogs**	**32**	**30**	**16**	95.06 ± 18.12 (74.11-152.28)	34.78 (33.06)
Bathinda ward 11	Bathinda	Adult Male	14	18	8	50 ± 14.94 (36.36-106.37)	36 (18)

	Adult female	16	19	10	50.62 ± 8.67 (40.66-78.14)	30.86 (15.62)
	Total adult	30	37	18	100.35 ± 17.27 (79.83-153.67)	33.23 (33.35)
	Young male	7	4	3	16.33 ± 9.57 (11.51-67.05)	32.65 (5.33)
	Young female	4	5	3	12 ± 5.1 (9.31 -38.44)	25 (3)
	Total young	11	9	6	28.17 ± 11.14 (21.09-80.93)	28.99 (8.17)
	Total male	21	22	11	66.27 ± 14.2 (50.73-113.08)	35.12 (23.27)
	Total female	20	24	13	62.48 ± 12.67 (49.47-106.41)	29.58 (18.48)
	**Total dogs**	**41**	**46**	**24**	128.34 ± 15.39 (107.41-170.76)	32.21 (41.34)
**Total dogs in rural area**	Adult Male	271	261	191	684.2 ± 24.63 (643.05-740.59)	22.24 (152.2)
	Adult female	212	213	159	536.25 ± 16.51 (508.31-573.57)	20.75 (111.25)
	Total adult	483	474	350	1220.2 ± 29.56 (1168.3-1284.8)	21.57 (263.18)
	Young male	63	66	44	170.05 ± 12.94 (151.46-204.04)	24.14 (41.05)
	Young female	68	66	46	176.09 ± 13 (157.29-210.05)	23.9 (42.09)
	Total young	131	132	90	346.14 ± 22.01 (312.91-401.47)	24.02 (83.14)

	Total male	334	327	235	854.06 ± 24.67 (811.44-908.75)	22.6 (193.06)
	Total female	280	279	205	711.82 ± 22.94 (673.06-763.77)	21.47 (152.82)
	Total dogs	614	606	440	1565.7 ± 34 (1505.2-1639)	22.08 (345.68)
	**Mean dogs per village**				**71.17 ± 1.54 (68.4-74.5)**	
**Total dogs in urban area**	Adult Male	164	169	87	506.9 ± 28.18 (459.84-571.42)	34.31 (173.9)
	Adult female	137	129	80	374.11 ± 24.36 (335.89-433.23)	28.9 (108.11)
	Total adult	301	298	167	878.38 ± 37.77 (813.61-962.7)	31.81 (279.38)
	Young male	51	46	27	142.37 ± 16.67 (119.59-188.14)	31.87 (45.37)
	Young female	45	52	30	134.41 ± 13.46 (115.88-171.12)	27.83 (37.41)
	Total young	96	98	57	276.32 ± 21.54 (243.71-330.32)	29.79 (82.32)
	Total male	215	215	114	648.98 ± 38.93 (584.98-739.42)	33.74 (218.98)
	Total female	182	181	110	508.48 ± 29.33 (461.38-578.12)	28.61 (145.48)
	Total dogs	397	396	224	1154.3 ± 46.13 (1074.6-1256.6)	31.3 (361.34)
	**Mean dogs**				**115.4 ± 4.61 (107.4-**	

	**per ward**				**125.7)**	
**Total dogs in rural & urban area**						
	Adult Male	435	430	278	1174.9 ± 38.46 (1108.2-1259.8)	26.37 (309.86)
	Adult female	349	342	239	904.71 ± 27.54 (857.17-965.84)	23.62 (213.71)
	Total adult	784	772	517	2078.01 ± 43.39 (1999.7-2170.2)	25.12 (522.01)
	Young male	114	112	71	310.03 ± 17.47 (282.51-352.25)	25.49 (79.03)
	Young female	113	118	76	310.03 ± 17.47 (282.51-352.25)	25.49 (79.03)
	Total young	227	230	147	620.44 ± 25.32 (577.85-678.04)	26.34 (163.44)
	Total male	549	542	349	1485.4 ± 41.71 (1411.7-1576)	26.55 (394.39)
	Total female	462	460	315	1214.4 ± 35.25 (1153.1-1292.1)	24.08 (292.42)
	Total dogs	1011	1002	664	2698.2 ± 50.86 (2605.3-2805.3)	25.39 (685.17)
